# Glutathione Peroxidase in Stable Chronic Obstructive Pulmonary Disease: A Systematic Review and Meta-analysis

**DOI:** 10.3390/antiox10111745

**Published:** 2021-10-30

**Authors:** Elisabetta Zinellu, Angelo Zinellu, Maria Carmina Pau, Barbara Piras, Alessandro G. Fois, Sabrina Mellino, Ciriaco Carru, Arduino A. Mangoni, Pietro Pirina

**Affiliations:** 1 Clinical and Interventional Pulmonology, University Hospital Sassari (AOU), 07100 Sassari, Italy; elisabetta.zinellu@aousassari.it (E.Z.); agfois@uniss.it (A.G.F.); 2 Department of Biomedical Sciences, University of Sassari, 07100 Sassari, Italy; azinellu@uniss.it (A.Z.); s.mellino@studenti.uniss.it (S.M.); carru@uniss.it (C.C.); 3 Department of Medical, Surgical and Experimental Sciences, University of Sassari, 07100 Sassari, Italy; mcpau@uniss.it (M.C.P.); barbara.piras@aousassari.it (B.P.); 4 Department of Clinical Pharmacology, College of Medicine and Public Health, Flinders Medical Centre, Flinders University, Bedford Park, SA 5042, Australia; arduino.mangoni@flinders.edu.au

**Keywords:** chronic obstructive pulmonary disease, oxidative stress, antioxidant defense systems, glutathione peroxidase

## Abstract

Chronic obstructive pulmonary disease (COPD) is a progressive disease that is characterized by a state of persistent inflammation and oxidative stress. The presence of oxidative stress in COPD is the result of an imbalance between pro-oxidant and antioxidant mechanisms. The aim of this review was to investigate a possible association between glutathione peroxidase (GPx), a key component of antioxidant defense mechanisms, and COPD. A systematic search for relevant studies was conducted in the electronic databases PubMed, Web of Science, Scopus, and Google Scholar, from inception to June 2021. Standardized mean differences (SMDs) were used to express the differences in GPx concentrations between COPD patients and non-COPD subjects. Twenty-four studies were identified. In 15 studies assessing whole blood/erythrocytes (GPx isoform 1), the pooled results showed that GPx concentrations were significantly lower in patients with COPD (SMD = −1.91, 95% CI −2.55 to −1.28, *p* < 0.001; moderate certainty of evidence). By contrast, in 10 studies assessing serum/plasma (GPx isoform 3), the pooled results showed that GPx concentrations were not significantly different between the two groups (very low certainty of evidence). The concentration of GPx-1, but not GPx-3, is significantly lower in COPD patients, suggesting an impairment of antioxidant defense mechanisms in this group.

## 1. Introduction

Chronic obstructive pulmonary disease (COPD) is an inflammatory disease characterized by persistent airflow limitation due to airway obstruction and/or lung tissue damage [1]. With a global prevalence of 13.1%, COPD is the third leading cause of death worldwide [2,3]. Oxidative stress and inflammation are considered key drivers of the pathophysiology of COPD [4,5,6]. The lungs are particularly exposed to environmental insults, such as tobacco smoke and air pollutants, that represent important sources of reactive oxygen species (ROS). The latter directly promote lung damage, resulting from alterations of DNA, lipids, carbohydrates, and proteins, as well as activate local inflammatory responses which contribute to the development and progression of COPD [6]. ROS can also activate epithelial cells and macrophages as well as facilitate the recruitment of neutrophils, monocytes, and lymphocytes. Recruited inflammatory cells become activated and then generate further ROS, enhancing the pro-oxidant burden [7,8]. These events lead to a state of sustained inflammation and chronic oxidative stress. Moreover, it has been reported that traffic-related air pollution increases airway inflammation which induces the expression of inflammatory factors through the activation of the NF-κB signaling pathway [9]. Environmental exposure can also induce oxidative stress through a disruption in the expression of micro-RNA, short sequences of non-coding RNA molecules which are involved in the regulation of gene expression [10]. Increased oxidative stress in COPD patients, which has been convincingly demonstrated with various biomarkers, reflects both an increase in oxidant molecules and a decrease in antioxidant defense mechanisms [11,12]. Specifically, the antioxidant defense mechanisms are overwhelmed in the presence of excess ROS. These antioxidant defenses mainly consist of non-enzymatic molecules, such as vitamins, glutathione, and protein thiols, most notably albumin, and enzymatic molecules, such as superoxide dismutase, catalase and glutathione peroxidase. Among these enzymes, glutathione peroxidase (GPx) has received particular attention in COPD. GPx catalyzes the reduction of lipid hydroperoxides into their corresponding alcohols and the reduction of hydrogen peroxide into water, using glutathione as reducing substrate [13,14]. The GPx family includes eight isoforms with different expression and antioxidant properties in individual tissues [13]. Only the first four, all of which are selenoproteins, have been well characterized: GPx-1, the predominant isoform, is ubiquitously expressed in the cytosol and mitochondria; GPx-2 is localized in the gastrointestinal epithelium; GPx-3 is the only member of the GPx family that is present in the extracellular compartment; GPx-4 (phospholipids hydroperoxide GPx) has a different subcellular localization and protects the membrane against lipid peroxidation [13]. The assessment of GPx activity in COPD patients may be useful to detect an impaired antioxidant defense system in this group. Several studies have reported GPx activity in the blood of stable COPD patients and non-COPD subjects, however, the results were not always concordant or significant. Therefore, we sought to further investigate this issue by performing a comprehensive assessment of all published studies by means of systematic review and meta-analysis. We hypothesized that the presence of COPD would be associated with a significant reduction in GPx concentrations in the blood.

## 2. Materials and Methods

### 2.1. Search Strategy, Eligibility Criteria, and Study Selection

A systematic search was conducted in the electronic databases PubMed, Web of Science, Scopus, and Google Scholar, from inception to June 2021, using combinations of the following terms: “Glutathione Peroxidase” or “GPx” or “GSH-PX” and “Chronic Obstructive Pulmonary Disease” or “COPD”. Two investigators independently reviewed the full text of the articles once their abstracts were deemed relevant. Eligibility criteria were: (i) the assessment of GPx in blood, erythrocytes, plasma or serum; (ii) a comparison of adult human subjects with COPD and non-COPD (case–control design); (iii) a sample size of ≥ 10 patients with COPD; (iv) English language and (v) full-text available. The references of the retrieved articles were also searched to identify additional studies. To evaluate the risk of bias, the Joanna Briggs Institute (JBI) Critical Appraisal Checklist was used, with scores ≥ 5, 4, and < 4 indicating low, moderate, and high risk, respectively [15]. We assessed the certainty of evidence following the Grades of Recommendation, Assessment, Development and Evaluation (GRADE) Working Group system. GRADE addresses the following domains: study design, the risk of bias, unexplained heterogeneity, indirectness of evidence, imprecision of the results, effect size, and the probability of publication bias [15].

### 2.2. Statistical Analysis

Since different units of measurement (U/L, U/gHb or U/mg protein) were used, standardized mean differences (SMDs) were calculated to build forest plots of continuous data and to express the differences in GPx concentrations in COPD patients vs. non-COPD subjects. A *p*-value < 0.05 was considered statistically significant, and 95% confidence intervals (CIs) were reported. If necessary, the mean and standard deviation values were extrapolated from median and interquartile ranges or medians and ranges, as reported by Wan et al. [16] and by Hozo et al. [17], respectively, or from graphs generated by using the Graph Data Extractor software (San Diego, CA, USA).

To test the heterogeneity of SMD across studies the Q-statistic (the significance level at *p* < 0.10) was used. We used fixed-effects and random-effects models for a pooled analysis with low heterogeneity (I^2^ statistic < 50% or *p*-value < 0.1) and high heterogeneity (I^2^ statistic > 50% or *p*-value ≤ 0.1), respectively [18,19]. A sensitivity analysis was also performed to evaluate the robustness of the pooled effect estimates by sequentially excluding each study and repeating the meta-analysis after each iteration [20].

The Begg’s adjusted rank correlation test and the Egger’s regression asymmetry test, at the *p* < 0.05 level of significance, were also performed to evaluate the presence of publication bias [21,22]. The latter was further investigated using the Duval and Tweedie “trim-and-fill” method [23]. Univariate meta-regression analyses were conducted to investigate the presence of associations between the effect size and the following parameters: age, gender, FEV1 (forced expiratory volume in the 1st second), FEV1/FVC (forced expiratory volume in in the 1st second /forced vital capacity), and the guidelines used for diagnosis (GOLD vs. ATS guidelines). Information regarding missing data in the original articles was not queried upon to the authors. This study followed the guidelines for systematic reviews which are illustrated in the PRISMA Statement [24]. Statistical analyses were performed using Stata 14 (STATA Corp., College Station, TX, USA). The study protocol was registered in the International Prospective Register of Systematic Reviews (PROSPERO registration number: CRD42021276524).

## 3. Results

### 3.1. Systematic Research

**Figure 1:**
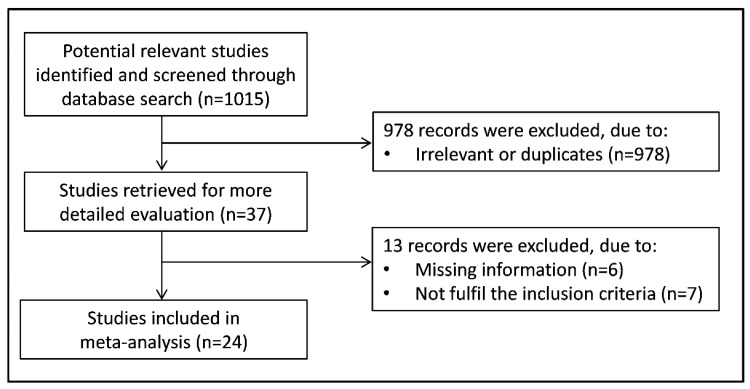
Flow chart of study selection.

Figure 1 shows the flow chart depicting the screening process. We identified 1,015 articles from the database search. After screening the abstracts and titles of the studies, 37 were selected for full-text evaluation. Of these, 13 were further excluded, either because of missing information or they did not fulfil the inclusion criteria. Finally, 24 studies were included in the meta-analysis [25,26,27,28,29,30,31,32,33,34,35,36,37,38,39,40,41,42,43,44,45,46,47,48]. A total of 2214 COPD patients (mean age 60 years, 74% male), and 1608 non-COPD subjects (mean age 55 years, 71% male) were evaluated. The characteristics of the retrieved studies, published between 1994 and 2019, are described in Table 1.

### 3.2. Meta-Analysis of Whole Blood/Erythrocyte GPx Concentrations

#### 3.2.1. Study Characteristics

Fifteen studies on 1329 COPD patients (mean age 60 years, 79% male) and 814 non-COPD subjects (mean age 54 years, 71% male) were identified [25,26,27,28,29,30,31,32,33,34,35,36,37,38,39]. COPD was diagnosed according to the Global Obstructive Lung Disease (GOLD) guidelines in 12 studies [25,26,28,29,31,32,33,34,35,36,37,38], and the American Thoracic Society/European Respiratory Society (ATS/ERS) guidelines in three [27,30,39]. Thirteen studies assessed erythrocytes [25,26,27,28,29,30,31,32,33,34,35,37,39] whereas the remaining two assessed whole blood [36,38].

#### 3.2.2. Risk of Bias

The risk of bias was considered low in seven studies [26,27,28,29,30] and moderate in the remaining eight [25,31,33,34,35,36,38,39] (Table 2).

#### 3.2.3. Results of Individual Studies and Syntheses

**Figure 2:**
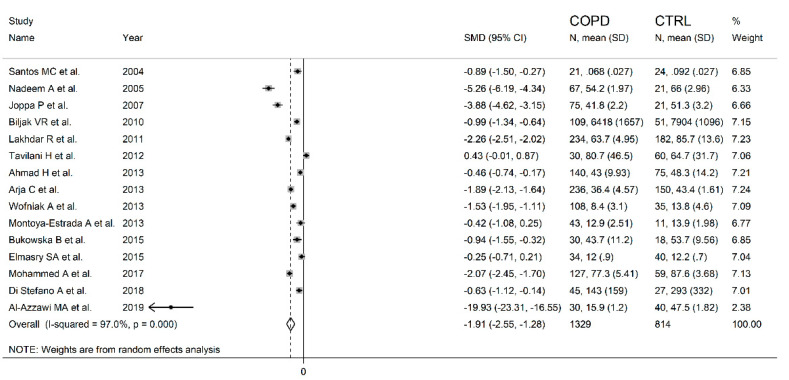
Forest plot of studies examining blood/erythrocytes GPx values of COPD and non-COPD.

**Figure 3:**
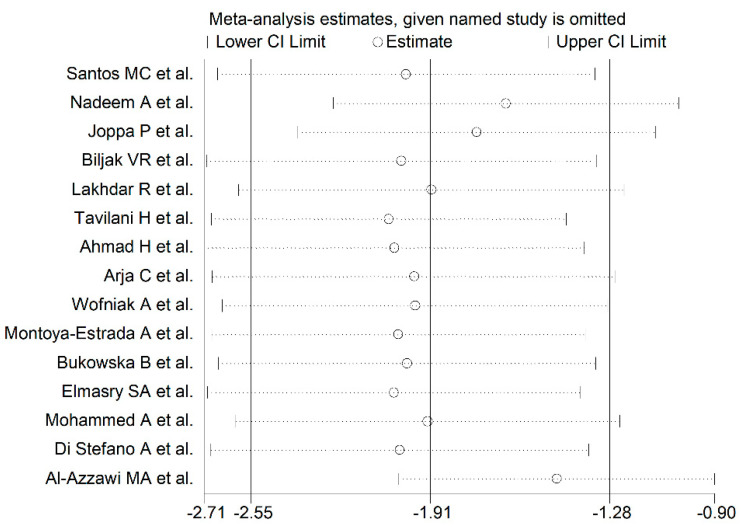
Sensitivity analysis of the association between blood/erythrocytes GPx and COPD disease. For each study, the displayed effect size (hollow circles) corresponds to an overall effect size computed from a meta-analysis excluding that study.

**Figure 4:**
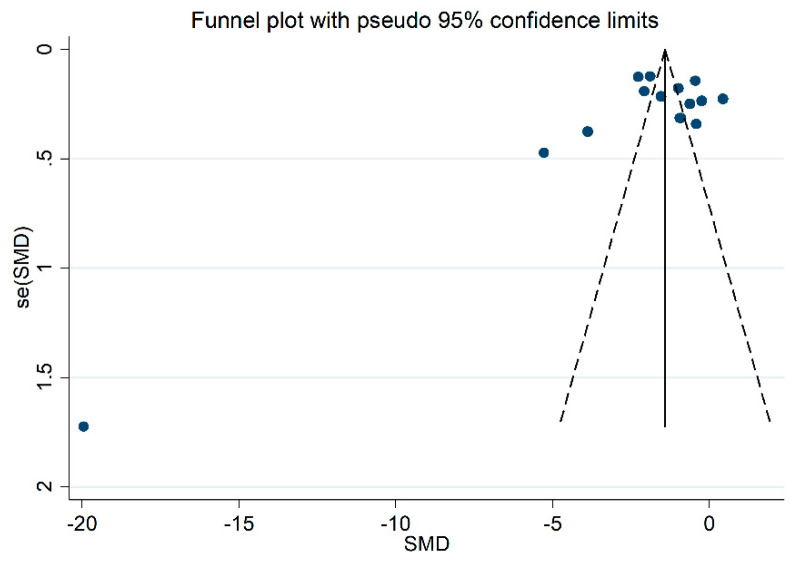
Funnel plot of the 15 retrieved studies evaluating the association between blood/erythrocytes GPx concentration and COPD disease.

The forest plot for the blood GPx concentrations in COPD patients and non-COPD subjects is reported in Figure 2. In 14 studies [25,26,27,28,29,31,32,33,34,35,36,37,38,39], COPD patients had lower blood GPx concentrations when compared to non-COPD subjects (mean difference range, −0.42 to −19.93), however, the difference was statistically significant in only two studies [34,36]. Extreme heterogeneity between studies was observed (I^2^ = 97.0%, *p* < 0.001). Thus, random-effects models were used. Overall, the pooled results showed that blood GPx concentrations were significantly lower in COPD patients (SMD= −1.91, 95% CI −2.55 to −1.28; *p* < 0.001). Sensitivity analysis showed that the corresponding pooled SMD values were not altered when any single study was sequentially omitted (effect size range, between −2.04 and −1.46, Figure 3). However, funnel plot analysis showed that the study by Al-Azzawy et al. [39] influenced graph symmetry which had a possible effect on the magnitude of the results (Figure 4). After removing this study, the SMD was attenuated but remained significant (SMD = −1.46, 95% CI −2.02 to −0.90, *p* < 0.001) with persistent, extreme heterogeneity (I^2^ = 96.2%, *p*  <  0.001).

#### 3.2.4. Publication Bias

**Figure 5:**
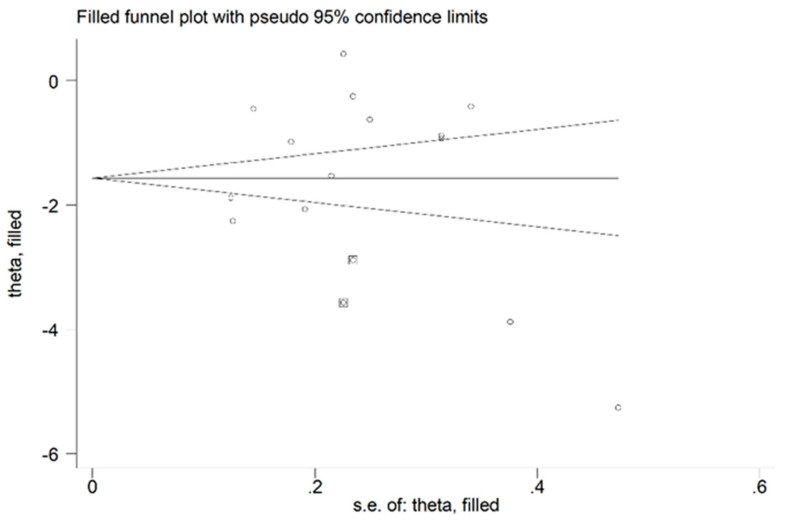
Funnel plot of studies investigating the association between blood/erythrocytes GPx concentration and COPD disease after trimming and filling. Dummy studies and genuine studies are represented by enclosed circles and free circles, respectively.

There was no publication bias, after removing the study by Al-Azzawy et al. [39] (Begg’s test, *p* = 0.74; Egger’s test, *p* = 0.94). The “trim-and-fill” method identified two potential missing studies to be added to the left side of the funnel plot to ensure symmetry (Figure 5). The adjusted SMD was further increased as a result (SMD = −1.69, 95% CI −2.26 to −1.12, *p* < 0.001).

#### 3.2.5. Meta-Regression and Sub-group Analysis

**Figure 6:**
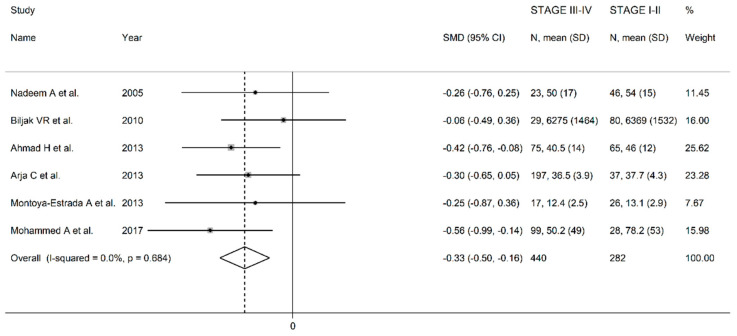
Forest plot of studies examining erythrocyte GPx concentrations of COPD patients in stage I-II vs stage III-IV.

In univariate meta-regression, no significant associations were observed between the effect size and age (*t* = −1.89, *p* = 0.10), gender (*t* = 0.78, *p* = 0.46), FEV1 (*t* = 0.62, *p* = 0.55), FEV1/FVC (*t* = −0.50, *p* = 0.63), or specific guideline used (t = −0.20, *p* = 0.85). In the sub-group analysis, the pooled SMD value in studies which measured GPx in whole blood (SMD = −0.43, 95% CI −0.80 to −0.06, *p* = 0.023; I^2^ = 18.4%, *p* = 0.268) was non-significantly higher (t = 1.07, *p* = 0.31) than that observed in studies which assessed GPx in erythrocytes (SMD= −1.64, 95% CI −2.25 to −1.03, *p* < 0.001; I^2^ = 96.4%, *p* < 0.001). The search for more homogeneous study sub-groups, according to diagnostic guidelines, matrix type and continent, led to the identification of four studies conducted in Europe which used the GOLD guidelines and assessed erythrocytes [25,28,33,35]. The effect size was still significant (SMD= −1.12, 95% CI −1.43 to −0.81, *p* < 0.001) with a substantially lower heterogeneity (I^2^ = 41.4%, *p* = 0.16). In order to evaluate the relationship between the effect size and disease severity we performed a further meta-analysis in a sub-group of six studies which reported the erythrocyte GPx concentrations in groups with different disease severities (GOLD stage I-II vs III-IV) [26,28,31,32,34,37]. The forest plot for the GPx concentrations in mild/moderate vs severe/very severe COPD patients is reported in Figure 6. In all studies, the GPx concentrations were lower in patients with severe disease (mean difference range −0.56 to −0.06), with a significant difference in two studies [31,37]. The pooled results showed that GPx concentrations were significantly lower in patients with more severe disease (SMD = −0.33; 95% CI −0.50 to −0.16, *p* < 0.001; I^2^ = 0.0%, *p* = 0.68).

#### 3.2.6. Certainty of Evidence

The initial level of certainty for the blood/erythrocyte GPx SMD values was considered low because of the observational nature of the selected studies (rating 2, ⊕⊕⊝⊝). After considering the presence of a moderate risk of bias in 8 out of 15 studies (a serious limitation, downgrade one level), a generally extreme heterogeneity that was partly explained by specific diagnostic guidelines, the matrix type, and continent (no rating change required), the lack of indirectness (no rating change required), the relatively low imprecision (relatively narrow confidence intervals without threshold crossing, no rating change required), the relatively large effect size (SMD −1.91, upgrade one level), and the absence of publication bias (upgrade one level), the overall level of certainty was considered moderate (rating 3, ⊕⊕⊕⊝).

### 3.3. Meta-analysis of Serum/Plasma GPx Concentrations

#### 3.3.1. Study Characteristics

Ten studies in 885 COPD patients (mean age 60 years, 66% male) and 794 non-COPD subjects (mean age 55 years, 70% male) were identified [26,40,41,42,43,44,45,46,47,48]. A COPD diagnosis was made according to the Global Obstructive Lung Disease (GOLD) guidelines in 8 studies [26,42,43,44,45,46,47,48], and the American Thoracic Society/ European Respiratory Society (ATS/ERS) guidelines in two [40,41]. Plasma was analyzed in 8 studies [26,41,42,44,45,46,47,48], whereas serum was assessed in two [40,43].

#### 3.3.2. Risk of Bias

The risk of bias was considered low in seven studies [26,41,43,44,45,46,47], moderate in one [40] and high in the remaining two [42,48] (Table 2).

#### 3.3.3. Results of Individual Studies and Syntheses

**Figure 7:**
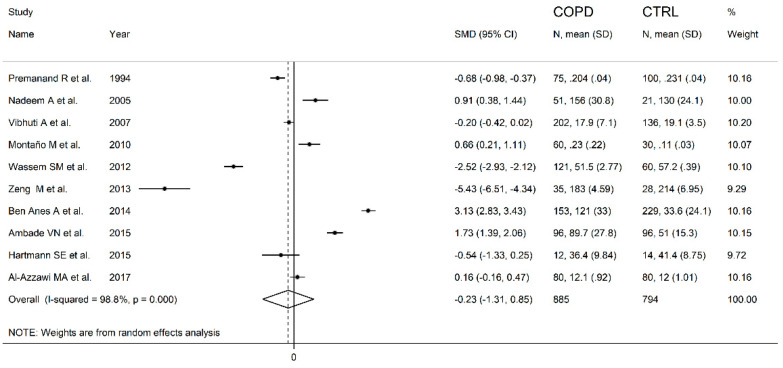
Forest plot of studies examining the serum/plasma GPx values of COPD and non-COPD.

**Figure 8:**
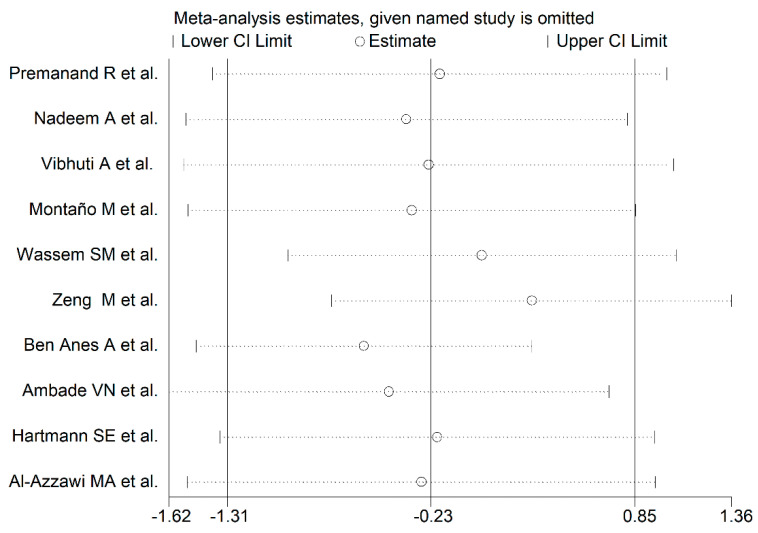
Sensitivity analysis of the association between serum/plasma GPx and COPD disease. For each study, the displayed effect size (hollow circles) corresponds to an overall effect size computed from a meta-analysis which excluded that study.

The forest plot for the serum/plasma GPx concentrations in COPD patients and non-COPD subjects is described in Figure 7. In five studies [40,41,43,44,47], COPD patients had lower serum GPx concentrations when compared to non-COPD subjects (mean difference range, −5.43 to −0.20), and this difference was statistically significant in three studies [40,43,44]. In the remaining five studies [26,42,45,46,48], COPD patients had higher serum GPx concentrations (mean difference range, 0.16 to 3.13), and this difference was statistically significant in four studies [26,42,45,46]. Extreme heterogeneity between studies was observed (I^2^ = 98.8%, *p* < 0.001). Thus, random-effects models were used. Overall, the pooled results showed that the serum/plasma GPx concentrations were not significantly different between the two groups (SMD= −0.23, 95% CI −1.31 to 0.85, *p* = 0.67). The effect size was not substantially altered (range between −0.59 and 0.30, Figure 8) after sequentially removing individual studies.

#### 3.3.4. Publication Bias

There was no publication bias according to the Begg’s (*p* = 0.59) and Egger’s (*p* = 0.46) tests, or the “trim-and-fill method”.

#### 3.3.5. Meta-regression and Sub-group Analysis

Sub-group analysis showed that the pooled SMD value for the studies measuring GPx in serum (SMD = −1.59, 95% CI −3.41 to −0.22, *p* = 0.084; I^2^ = 98.0%, *p* < 0.001) was non-significantly lower (*t* = 0.91, *p* = 0.39) than that observed in the studies assessing plasma (SMD = 0.12, 95% CI −1.00 to 1.25, *p* = 0.83; I^2^ = 98.6, *p* < 0.001). In addition, the pooled SMD value for the studies using the GOLD guidelines (SMD = −0.20, 95% CI −1.67 to 1.27, *p* = 0.79; I^2^ = 99.0%, *p* < 0.001) was similar (*t* = 0.12, *p* = 0.91) to that of the studies using the ATS guidelines (SMD = −0.43, 95% CI −0.89 to 0.04, *p* = 0.07, I^2^ = 83.4, *p* = 0.014).

#### 3.3.6. Certainty of Evidence

The initial level of certainty for serum/plasma GPx SMD values was considered low because the selected studies were observational (rating 2, ⊕⊕⊝⊝). After considering the presence of a low risk of bias in 7 out of 10 studies (no rating change required), the generally extreme and unexplained heterogeneity (a serious limitation, downgrade one level), the lack of indirectness (no rating change required), the relatively high imprecision (relatively narrow confidence intervals with threshold crossing, downgrade one level), the relatively small effect size (SMD −0.23, downgrade one level), and the absence of publication bias (upgrade one level), the overall level of certainty was considered downgraded to very low (rating 0, ⊝⊝⊝⊝).

## 4. Discussion

This meta-analysis provides a critical appraisal of the association between blood GPx concentrations and the presence of COPD. Twenty-four case–control studies were included. and further analyzed according to whether the assessment was performed in whole blood/erythrocytes or serum/plasma.

The results showed that the GPx concentrations in whole blood or erythrocytes were significantly lower in COPD patients when compared to non-COPD subjects. The observed pooled SMD value (−1.91) indicated the presence of a large effect size [49], even after removing the study by Al-Azzawy [39] that appeared to influence the funnel plot symmetry (−1.46). Although a substantial heterogeneity between studies was observed, the sensitivity analysis showed that the pooled SMD value was not altered when individual studies were sequentially discarded. Furthermore, the Begg’s and Egger’s tests revealed the absence of a publication bias. The meta-regression analysis did not find associations between the effect size and age, gender or lung function parameters. The sub-group analysis identified four studies that were homogeneous regarding the diagnostic guidelines, matrix type, and continent. In this subgroup, the effect size confirmed that the GPx concentrations were significantly lower in COPD patients, but with a substantially lower heterogeneity between studies. This suggests that these factors can influence the observed heterogeneity. However, additional potential heterogeneity could also depend on other unreported factors, such as differences in sample handling and analytical procedure, or other inter-individual differences. Moreover, six studies allowed us to further evaluate the relationship between effect size and disease severity, which indicated that erythrocyte GPx concentrations were significantly lower in the patients with more severe disease.

In contrast to the assessment of whole blood and erythrocytes, the studies that assessed GPx in serum or plasma showed conflicting results. This could be due to differences in analytical approaches, age, gender, diet, or lifestyle, which might influence per se the concentration of antioxidant molecules. Therefore, the overall SMD value did not significantly differ between the two groups. There was a substantial heterogeneity between the studies, however the pooled SMD value was not altered when any single study was sequentially removed.

The observed differences in the pooled SMD between the two meta-analyses highlight the importance of the specific biological matrices GPx isoforms. In blood, two isoforms are mainly represented, the intracellular isoform GPx-1, which is ubiquitously expressed in the cytosol, and the extracellular GPx-3, which is actively released into the plasma where it is primarily present as a glycosylated protein [13,50]. Both isoforms are homo-tetramers containing a selenocysteine in their active site, which catalyzes the reduction of hydrogen peroxide or organic hydroperoxides to water or corresponding alcohols [51]. GPx-1, the first selenoprotein identified and characterized as an erythrocytic enzyme, protects hemoglobin from oxidative damage [52]. Red blood cells are normally exposed to high oxygen concentrations, which promote the production of ROS. Our meta-analysis has shown for the first time that GPx-1, but not GPx-3, is significantly lower in COPD patients when compared to non-COPD subjects, and in COPD patients with more severe disease when compared to those with milder forms, which further supports the pathophysiological role of oxidative stress in this disabling condition. The significant reduction of the erythrocytic isoform of GPx may be partly explained by a significant exposure of this type of cell to oxidative stress and an impaired antioxidant system. It has been shown that GPx-1 expression is diminished by selenium deficiency both in vitro and in vivo studies [14]. It is also known that patients affected by COPD often exhibit nutritional deficiencies, including selenium deficiency [25]. This could contribute to the reduced GPx-1 activity observed in this disease. Moreover, the diminished activity of this enzyme, which uses GSH as co-substrate, may also be the consequence of the reduced GSH concentrations that are reported in COPD [53]. Finally, a reduction in GPx-1 expression has been also described in the airway epithelial cells in COPD patients due to accelerated mRNA degradation [54]. Thus, a more thorough evaluation of this important component of the antioxidant defense system may provide useful insights into its role in COPD development and progression, and as a marker of therapeutic response.

## 5. Conclusions

This meta-analysis had some limitations, in particular the presence of high heterogeneity, and the lack of sub-studies on the relation between GPx expression and clinical parameters, such as smoking habits or other environmental exposure. Furthermore, the number of studies included was limited to those written in English. On the other hand, strengths of our study include the assessment of individual matrix types, hence isoforms, and a comprehensive evaluation of the certainty of evidence for the SMD values. Whilst the presence of extreme heterogeneity might curtail the generalizability of our findings, we also identified that the use of specific COPD diagnostic guidelines, matrix types, and geographical areas are important contributors to such heterogeneity. Our findings support the presence of an impaired antioxidant defense system in COPD. The identification of GPx-1 as a potential biomarker of oxidative stress in COPD warrants longitudinal studies to determine its prognostic role in terms of disease progression and mortality, and to investigate the effects of specific antioxidant therapies in these patients.

## Figures and Tables

**Table 1:** Summary of the studies on non-COPD subjects vs COPD patients included in the meta-analysis.

		Non-COPD				COPD			
First Author Year, Country	Matrix Type	n	Age Mean	Gender (M/F)	GPx Mean ± SD	n	Age Mean	Gender (M/F)	GPX Mean ± SD
WHOLE BLOOD/ERYTHROCYTES									
Santos MC et al. 2004, Portugal	Er	24	NR	NR	0.092 ± 0.027 U/g Hb	21	NR	NR	0.068 ± 0.027 U/g Hb
Nadeem A et al. 2005, India	Er	21	NR	NR	65.96 ± 13.56 mU/g Hb	67	NR	NR	54.17 ± 16.12 mU/g Hb
Joppa P et al. 2007, Slovakia	Er	21	48	9/12	51.30 ± 14.66 U/g Hb	75	65	58/17	41.80 ± 18.05 U/g Hb
Biljak VR et al. 2010, Croatia	Er	51	52	21/30	7904 ± 1096 U/L	109	71	82/27	6418 ± 1657 U/L
Lakhdar R et al. 2011, Tunisia	Er	182	56	173/9	85.70 ± 13.61 U/g Hb	234	62	222/12	63.66 ± 4.95 U/g Hb
Tavilani H et al. 2012, Iran	Er	60	67	NR	64.7 ± 31.70 U/g Hb	30	66	NR	80.74 ± 46.50 U/g Hb
Ahmad H et al. 2013, India	Er	75	42	53/22	48.32 ± 14.20 U/g Hb	140	45	111/29	43.04 ± 9.93 U/g Hb
Arja C et al. 2013, India	Er	150	61	NR	43.63 ± 1.61 U/g Hb	236	63	NR	36.36 ± 4.57 U/g Hb
Wozniak A et al. 2013, Poland	Er	35	45	19/16	13.8 ± 46 U/g Hb	108	49	61/47	8.4 ± 3.1 U/g Hb
Montoya-Estrada A et al. 2013, Mexico	Er	11	61	1/10	13.90 ± 1.98 mU/mg protein	43	69	30/13	12.89 ± 2.51 mU/mg protein
Bukowska B et al. 2015, Poland	Er	18	NR	NR	53.66 ± 9.56 mU/g Hb	30	NR	NR	43.70 ± 11.20 mU/g Hb
Elmasry SA et al. 2015, Egypt	WB	40	54	31/9	12.2 ± 0.7 U/mL	34	55	27/7	12 ± 0.9 U/mL
Mohammed A et al. 2017, India	Er	59	51	38/21	63.77 ± 3.38 U/mg protein	127	60	98/29	59.43 ± 5.63 U/mg protein
Di Stefano A et al. 2018, Italy	WB	27	NR	12/15	293 ± 332 U/mL	45	NR	39/6	143 ± 159 U/mL
Al-Azzawi MA et al. 2019, Egypt	Er	40	45	28/12	47.5 ± 1.82 U/mL	30	65	21/9	15.9 ± 1.2 U/mL
SERUM/PLASMA									
Premanand R et al. 1994, India	S	100	NR	60/40	0.231 ± 0.040 U/mL	75	NR	43/32	0.204 ± 0.040 U/mL
Nadeem A et al. 2005, India	P	21	NR	NR	129.9 ± 24.1 mU/g Hb	51	NR	NR	156.4 ± 30.8 mU/g Hb
Vibhuti A et al. 2007, India	P	136	50	110/26	19.1 ± 3.5 U/mL	202	59	160/42	17.9 ± 7.1 U/mL
Montaño M et al. 2010, Mexico	P	30	65	0/30	0.11 ± 0.03 U/mL	60	73	0/60	0.23 ± 0.22 U/mL
Wassem SM et al. 2012, India	S	60	38	46/14	57.21 ± 0.39 mU/mg protein	121	48	80/41	51.46 ± 2.77 mU/mg protein
Zeng M et al. 2013, China	P	28	69	23/5	214.2 ± 6.9 U	35	71	31/4	183.0 ± 4.6 U
Ben Anes A et al. 2014, Tunisia	P	229	58	NR	33.6 ± 24.1 U/ml	153	61	NR	121.3 ± 33.0 U/ml
Ambade VN et al. 2015, India	P	96	60	73/23	50.95 ± 15.30 U/L	96	68	73/23	89.73 ± 27.84 U/L
Hartmann SE et al. 2015, Canada	P	14	68	6/8	41.41 ± 8.75 U/ml	12	69	4/8	36.41 ± 9.84 U/ml
Al-Azzawi MA et al. 2017, Egypt	P	80	53	62/18	11.98 ± 1.01 mU/ml	80	55	58/22	12.13 ± 0.92 mU/ml

Er = Erythrocytes; Hb: Haemoglobin; NR = Not reported; P = Plasma; S = Serum; WB = Whole Blood

**Table 2:** The Joanna Briggs Institute critical appraisal checklist for analytical cross-sectional studies.

Study	Were the Criteria for Inclusion in the Sample Clearly Defined?	Were the Study Subjects and the Setting Described in Detail?	Was the Exposure Measured in a Valid and Reliable Way?	Were Objective, Standard Criteria Used for Measurement of the Condition?	Were Confounding Factors Identified?	Were Strategies to Deal with Confounding Factors Stated?	Were the Outcomes Measured in a Valid and Reliable Way?	Was Appropriate Statistical Analysis Used?	Risk of Bias
Santos et al.	No	Yes	Yes	Yes	No	No	Yes	No	Moderate
Nadeem et al.	Yes	Yes	Yes	Yes	No	No	Yes	No	Low
Joppa et al.	Yes	Yes	Yes	Yes	No	No	Yes	No	Low
Biljak et al.	Yes	Yes	Yes	Yes	No	No	Yes	No	Low
Lakhdar et al.	No	Yes	Yes	Yes	Yes	Yes	Yes	Yes	Low
Tavilani et al.	Yes	Yes	Yes	Yes	No	No	Yes	No	Low
Ahmad et al.	No	Yes	Yes	Yes	No	No	Yes	No	Moderate
Arja et al.	No	Yes	Yes	Yes	Yes	Yes	Yes	Yes	Low
Wozniak et al.	No	Yes	Yes	Yes	No	No	Yes	No	Moderate
Montoya et al.	No	Yes	Yes	Yes	No	No	Yes	No	Moderate
Bukowska et al.	No	Yes	Yes	Yes	No	No	Yes	No	Moderate
Elmasry et al.	No	Yes	Yes	Yes	No	No	Yes	No	Moderate
Mohammed et al.	Yes	Yes	Yes	Yes	Yes	Yes	Yes	Yes	Low
Di Stefano et al.	No	Yes	Yes	Yes	No	No	Yes	No	Moderate
Al-Azzawi et al.	No	Yes	Yes	Yes	No	No	Yes	No	Moderate
Premanand et al.	No	Yes	Yes	Yes	No	No	Yes	No	Moderate
Nadeem et al.	Yes	Yes	Yes	Yes	No	No	Yes	No	Low
Vibhuti et al.	No	Yes	Yes	Yes	Yes	Yes	Yes	Yes	Low
Montano et al.	No	No	Yes	No	No	No	Yes	No	High
Waseem et al.	Yes	Yes	Yes	Yes	No	No	Yes	No	Low
Zeng et al.	Yes	Yes	Yes	Yes	No	No	Yes	No	Low
Ben Anes et al.	Yes	Yes	Yes	Yes	Yes	Yes	Yes	Yes	Low
Ambade et al.	Yes	Yes	Yes	Yes	No	No	Yes	No	Low
Hartmann et al.	Yes	Yes	Yes	Yes	No	No	Yes	No	Low
Al-Azzawi et al.	No	Yes	No	No	No	No	Yes	No	High

## Data Availability

The data presented in this study are available in review.
